# Necrotizing fasciitis: strategies for diagnosis and management

**DOI:** 10.1186/1749-7922-2-19

**Published:** 2007-08-07

**Authors:** Korhan Taviloglu, Hakan Yanar

**Affiliations:** 1 Istanbul University, Istanbul Faculty of Medicine, Departmentof Surgery, Trauma & Surgical Emergency Service, 34210 Capa, Istanbul, Turkey

## Abstract

Necrotizing fasciitis (NF) is uncommon and difficult to diagnose, and it cause progressive morbidity until the infectious process is diagnosed and treated medically and surgically. The literature addressed NF contains confusing information, inaccurate bacteriologic data, and antiquated antibiotic therapy. A delay in diagnosis is associated with a grave prognosis and increased mortality. The main goal of the clinician must be to establish the diagnosis and initially treat the patient within the standard of care. This review is planned as a guide for the clinician in making an early diagnosis of NF and initiating effective medical and surgical therapy.

## Background

Necrotising fasciitis (NF) is a rare soft tissue infection, primarily involving the superficial fascia and resulting in extensive undermining of the surrounding tissues. If untreated, it is invariably fatal, and thus a high index of suspicion for the diagnosis is required [1]. Mortality remains still high in NF despite use of modern powerful antimicrobial drug regimens and advances in the care of the critically ill patients. Overall mortality ranges from 25 percent to 73 percent in the published literature [2-12]. The disease's manifestation can range from a fulminant presentation to a subtle and insidious development [13].

## Historical Perspective

In 1871, a Confederate army surgeon named Joseph Jones first described the disease during the Civil War. By 1918, the cause of the disease was identified as a bacterial infection. It was named "necrotizing fasciitis" in 1952, from *necrosis*, which means death of a portion of tissue, and *fascia*, which refers to the fibrous tissues that enclose and connect the muscles. Throughout the 19th and 20th centuries, cases of NF occurred only sporadically and usually remained restricted to military hospitals during wartime, although some civilian population outbreaks have also occurred. The Center for Disease Control and Prevention (CDC) reports that, worldwide, rates of NF increased from the mid-1980s to early 1990s. Increases in the rate and severity of NF are associated with increases in the prevalence of toxin-producing strains of *S. pyogenes *(M-1 and M-3 serotypes). In 1999, approximately 600 cases of NF were reported in the United States, according to the CDC [14].

## Diagnosis

The diagnosis of primary or idiopathic NF may be challenging because it occurs in the absence of a known causative factor or portal of entry for bacteria [11,15]. In most cases, NF occurs as a result of a known etiology, and classified as secondary NF. Bacterial entry occurs as a result of some precipitating events such as laceration, cut, abrasion, contusion, burn, bite, subcutaneous injection, or operative incision, that cause a break in the epidermidis. Secondary NF may also occur as a result of an occult infection such as a perforated viscus or as a complication of peri-rectal abscess or infected Bartholin cysts [16].

Primary or idiopathic NF, however, occurs in the absence of a known or identifiable etiologic factor. The underlying pathogenesis of idiopathic NF is still unknown. In terms of early diagnosis and management, it is important to consider that idiopathic NF exists, and that it is a distinct clinical entity.

The following symptoms of NF were compiled from the Center for Disease Control and Prevention and the National Necrotizing Fasciitis Foundation:

Early symptoms (usually within 24 hours):

1. Usually a minor trauma or other skin opening has occurred (the wound does not necessarily appear infected)

2. Some pain in the general area of the injury is present. Not necessarily at the site of the injury but in the same region or limb of the body

3. The pain is usually disproportionate to the injury and may start as something akin to a muscle pull, but becomes more and more painful

4. Flu like symptoms begin to occur, such as diarrhea, nausea, fever, confusion, dizziness, weakness, and general malaise

5. Dehydration

6. The biggest symptom is all of these symptoms combined. In general you will probably feel worse than you've ever felt and not understand why.

Advanced symptoms (usually within 3–4 days):

1. The limb, or area of body experiencing pain begins to swell, and may show a purplish rash

2. The limb may begin to have large, dark marks, that will become blisters filled with blackish fluid

3. The wound may actually begin to appear necrotic with a bluish, white, or dark, mottled, flaky appearance.

Critical symptoms (usually within 4–5 days):

1. Blood pressure will drop severely

2. The body begins to go into septic shock from the toxins the bacteria are giving off

3. Unconsciousness will occur as the body becomes too weak to fight off this infection.

DM is the leading predisposing factor in both idiopathic and secondary NF in our patient population. The mechanisms that has been suggested how DM could cause susceptibility to NF are: a) The peripheral sensory polyneuropathy experienced by diabetics may increase susceptibility to minor trauma. b) tissue hypoxia caused by diabetic vascular disease and the underlying immunodeficiency [17]. Even though there is substantial evidence indicating an important role of DM in the etiology of NF, its role as a predisposing factor for increased death rate is controversial. Some reports failed to show a significant relationship between mortality and DM in NF [9,18]. Interestingly, however, DM was determined as a significant factor associated with mortality in multivariate analysis in our study.

Immuno-competence has been claimed to be an important factor in the etiopathogenesis of NF [19]. Sudarsky et al reported that 91% of their patients with NF had some associated immunodeficiency [20]. These predisposing conditions are mainly DM, alcoholism, end-stage renal disease, malignancy, chemotherapy, malnutrition, corticosteroid use, multitrauma and the peripartum period [8,9,21].

## Prevention

The following is a list of recommendations to prevent the disease, as reported by the CDC.

• Good hand washing can prevent the spread of Group A Streptococcus (GAS) infection, especially after coughing, sneezing and before preparing food or eating.

• Patients with sore throats should be seen by a doctor.

• Patients with strep throat should stay home until 24 hours after their last antibiotic dose.

• Keeping the skin intact is important.

• Wounds should be cleaned and monitored for signs of infection (redness, swelling, drainage, pain).

• Keeping the skin intact is an important factor in preventing NF.

• Patients with an infected wound and fever should seek medical care.

## Treatment

The treatment for NF is a combination of surgical debridement, appropriate antibiotics and optimal oxygenation of the infected tissues.

NF a challenging and potentially lethal disease; early diagnosis is of principal importance and aggressive multidisciplinary treatment is mandatory. Early recognition and treatment by extensive debridement and antibiotics can prevent its fulminant course with a fatal outcome [8,9,11]. The priority in every case is to proceed to radical surgical debridement.

Once NF diagnosed all patients must be treated with immediate surgical debridement, and broad spectrum antibiotic combinations against the anaerobs, gram-negative and gram-positive bacilli, that were changed to other antibiotic combinations as determined according to the culture sensitivity of the microbial isolates and clinical course of the patients.

The microbiological isolates were polibacterial in the majority of the patients with either idiopathic or secondary NF in concordance with some recent studies [5,18,19]. Therefore, administration of broad-spectrum antibiotics appears to be important in the management of these patients.

Penicillin-clindamycin-gentamicin or ampicillin/sulbactam or nafcillin or cefazolin plus metronidazol combinations could be considered in the initial antibiotherapy of NF according to the physician's preference. If ESBL-producing *K. pneumoniae *susceptible to beta-lactam/beta-lactamase inhibitor combinations was identified (i.e, piperacillin/tazobactam, ticarcillin/clavulanate), one of these antibiotics or carbapenems in combination with aminoglycosides could be preferred. If *Clostridium perfringens *was suspected or identified, aqueous penicillin G (18 million units/day, if renal function is normal) was administered with or without hyperbaric oxygen-therapy.

Hyperbaric oxygen therapy (HBO) treatment increase tissue oxygenation in both healthy tissue and in the vicinity of infected tissue [22-24]. Hyperbaric oxygen-therapy was administered at 2.5 to 3.0 atmospheres for 90 min twice daily, following surgical debridement until no ongoing necrosis was evident in patients with clostridial infections. Critical care support was provided for patients with hemodynamic and ventilatory instability.

Elderly patients with underlying DM that have suspicious clinical findings of NF without any causative factors (e.g. trauma or operation) should be carefully be examined for the presence of idiopathic NF. The mortality rates are still very high in NF due to the severe sepsis that necessitates other interventions to overcome sepsis-related mortality.
